# Microbial communication leading to the activation of silent fungal secondary metabolite gene clusters

**DOI:** 10.3389/fmicb.2015.00299

**Published:** 2015-04-20

**Authors:** Tina Netzker, Juliane Fischer, Jakob Weber, Derek J. Mattern, Claudia C. König, Vito Valiante, Volker Schroeckh, Axel A. Brakhage

**Affiliations:** ^1^Department of Molecular and Applied Microbiology, Leibniz Institute for Natural Product Research and Infection Biology – Hans Knöll Institute, Jena, Germany; ^2^Department of Microbiology and Molecular Biology, Institute of Microbiology, Friedrich Schiller University Jena, Jena, Germany

**Keywords:** co-culture, secondary metabolite gene cluster activation, natural products, intermicrobial communication, posttranslational histone modifications, chromatin, acetyltransferases, mass spectrometry

## Abstract

Microorganisms form diverse multispecies communities in various ecosystems. The high abundance of fungal and bacterial species in these consortia results in specific communication between the microorganisms. A key role in this communication is played by secondary metabolites (SMs), which are also called natural products. Recently, it was shown that interspecies “talk” between microorganisms represents a physiological trigger to activate silent gene clusters leading to the formation of novel SMs by the involved species. This review focuses on mixed microbial cultivation, mainly between bacteria and fungi, with a special emphasis on the induced formation of fungal SMs in co-cultures. In addition, the role of chromatin remodeling in the induction is examined, and methodical perspectives for the analysis of natural products are presented. As an example for an intermicrobial interaction elucidated at the molecular level, we discuss the specific interaction between the filamentous fungi *Aspergillus nidulans* and *Aspergillus fumigatus* with the soil bacterium *Streptomyces rapamycinicus*, which provides an excellent model system to enlighten molecular concepts behind regulatory mechanisms and will pave the way to a novel avenue of drug discovery through targeted activation of silent SM gene clusters through co-cultivations of microorganisms.

## Introduction

**FIGURE 1:**
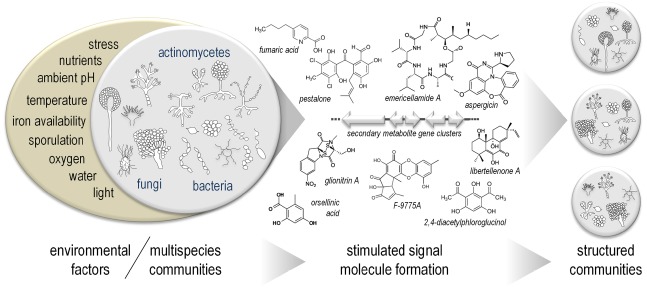
**Microorganismic multispecies communities form secondary metabolites that contribute to the stabilization/changes in these companionships.** In nature, microorganisms process signals from both abiotic and biotic environments. The latter represent secondary metabolites or natural products whose formation is often suppressed in pure cultures under standard conditions in the laboratory. Microbial mixed cultivation is therefore a suitable means to exploit their potential for natural product discovery and to study the molecular concepts behind the regulatory interactions.

Secondary metabolites (SMs) are low-molecular-mass organic compounds that, unlike primary metabolites, are not directly involved in growth, development or reproduction of the producing organism. Up until 2014 ∼170,000 natural products have been characterized from both marine and terrestrial organisms (Seyedsayamdost and Clardy, 2014; Chapman and Hall, 2015). Microorganisms are able to synthesize a large number of SMs, but the exact number is not known. Furthermore, mining of microbial genomes revealed the presence of numerous SM gene clusters, displaying a discrepancy between the number of putative genes involved in secondary metabolism and the known SMs in a single microbe (Bergmann et al., 2007; Sanchez et al., 2012; Craney et al., 2013). For example, the model fungus *Aspergillus nidulans* is potentially able to produce 32 polyketides, 14 non-ribosomal peptides and two indole alkaloids (Brakhage et al., 2008; Rank et al., 2010), with little more than 50% of the produced SMs being identified. Furthermore, SMs can be found in diverse environments and even chemical biogeographic distribution maps for biomedically valuable families of natural products in the environment have been created (Charlop-Powers et al., 2014). A number of these compounds have important pharmacological applications and are used as antibiotics/antibacterial drugs (Brakhage, 2013). Unfortunately, antibiotic resistance is spreading faster than the development of new antibiotics. As a consequence, there is the need for a constant provision of new compounds for the antibiotic development pipeline (Bbosa et al., 2014; Nathan and Cars, 2014). This is contrasted with a continuous rise in re-isolation of already known natural products (Strand et al., 2014). To manage this conflict, a more targeted natural product search is necessary. This effort directs SM research incrementally to a deeper understanding of the physiological relevance and ecological significance of SMs. It is generally accepted that in nature a substantial benefit to the SM producers must exist, simply arising from the fact that these very energy consuming biosynthetic pathways were maintained through evolution. An early postulated explanation for the role of SMs in nature was its function to defend the habitats of the producers by inhibiting the growth of its competitors (Davies, 1990; Brakhage et al., 2005; Galán et al., 2013). Another more recent hypothesis postulates an association between epibiotic predation and antibiotic production due to widespread predatory abilities in the genus *Streptomyces* (Kumbhar et al., 2014). At low, therefore non-inhibitory concentrations, such molecules are believed to function as signaling molecules (Aminov, 2009; Andersson and Hughes, 2014). This is supported by the assumption that over millions of years the evolution of SMs happened because microorganisms used them as chemical signals for communication between cells of the same species, different species (Figure 1) or between host cells, e.g., as endophytes in other microorganisms (Partida-Martinez and Hertweck, 2005) or plants (Brader et al., 2014).

As reported above, the majority of computationally identified SM gene clusters are silent under standard laboratory growth conditions. Successful methods to induce the formation of new metabolites include genetic engineering (Bergmann et al., 2007), mutagenesis, the OSMAC approach (Bode et al., 2002) or treatment with epigenetic modifiers (Cichewicz, 2010; Nützmann et al., 2011; Brakhage, 2013). In contrast to these classical methods, co-cultivation of bacteria–bacteria, fungi–fungi or of bacteria and fungi represent a naturally driven approach mimicking physiological conditions, involving competition and communication. Furthermore, co-cultures are highly relevant for drug research because they allow not only for the identification of new compounds, but can also monitor drug effects on synthetic microbial consortia. Up to now, various co-cultivation strategies have been applied. A summary with the focus on synthetic biology was given by Goers et al. (2014), while successful strategies with a special emphasis on SM formation in co-culture experiments were recently reviewed by Bertrand et al. (2014b).

The regulatory mechanisms of SM biosynthetic gene clusters are poorly understood. Unraveling both production conditions and signal transduction in nature, e.g., by identifying global regulators, will help to understand their function and support new possibilities to further explore SMs. Only a few examples on the gene regulatory network during SM formation in co-cultures have been reported. One is given by the specific interaction between *A. nidulans* and *Streptomyces rapamycinicus*. Thereby, activation of a fungal silent gene cluster and production of novel compounds was mediated by manipulating the chromatin-based regulation in the eukaryotic partner by the bacteria (Schroeckh et al., 2009; Nützmann et al., 2011). This review focuses on the communication between microorganisms, which has led to the activation of silent gene clusters and the formation of (novel) SMs by at least one of the involved species. Of particular focus is the bacteria-triggered activation of silent SM gene clusters in fungi and the role of chromatin remodeling in SM formation. Furthermore, methodical perspectives for the analysis of natural products are also discussed.

## Microbial Communication as an Inducer of Silent Secondary Metabolite Gene Clusters

Microbes and their SMs are known as one of the best resources for new drugs (Brakhage, 2013; Luo et al., 2014). Microorganisms form diverse multispecies communities within the natural environment. Here, they are subjected to intra- and interspecies interactions, which may result in beneficial or even harmful outcomes for the species involved. The real triggers leading to the activation of natural product biosynthesis in these communities are as diverse as the products themselves. They range from environmental signals, such as pH, carbon and nitrogen sources, to organisms living in the same habitat (Figure 1; Yu and Keller, 2005; Brakhage, 2013). Several recent reviews on mixed microbial cultivation and SMs have been published (Scherlach and Hertweck, 2009; Tarkka et al., 2009; Bertrand et al., 2014b; Marmann et al., 2014; Schroeckh et al., 2014), which clearly support co-cultivations of two (or even more) organisms on solid/liquid cultures as an adequate way to trace new metabolites. Additionally, such cultivations allow a tremendously enhanced production of already known natural compounds.

Starting in 1982, when Watanabe et al. (1982) discovered the formation of the antibacterial polyketide enacyloxin by *Gluconobacter* sp. W-315 during a co-cultivation with the fungi *Neurospora crassa* or *Aspergillus oryzae*, the number of publications dealing with mixed fermentations has drastically increased. The vast majority have been published within the last 5–7 years and nowadays co-cultivation of microbial species has turned into a key method in the discovery of new natural products with certain relevance to pharmaceutical or agricultural applications (Schroeckh et al., 2009; Brakhage, 2013; Moody, 2014).

The typical motivation for co-cultivation experiments is the identification of new bioactive compounds by unlocking cryptic SMs present in the genomes of the microorganisms in use. This has been shown for many microbial combinations, i.e., bacterium-bacterium, bacterium-fungus and fungus-fungus. Examples for SMs produced in fungus-fungus co-cultures are the acremostatins A-C, formed by *Acremonium* sp. in mixed culture with *Mycogone rosea* (Degenkolb et al., 2002), aspergicin, derived from a culture of two *Aspergillus* species (Zhu et al., 2011) or cyclo-(l-leucyl-trans-4-hydroxy-l-prolyl-d-leucyl-trans-4-hydroxy-l-proline), produced in the co-culture broth of two mangrove fungi *Phomopsis* sp. K38 and *Alternaria* sp. E33 (Li et al., 2014). Additionally, screening of fungal co-cultures in solid media led to the identification of an unusual long-distance growth inhibition between *Trichophyton rubrum* and *Bionectria ochroleuca* (Bertrand et al., 2013b). Analytical methods, such as LC-MS-based metabolomics (see below), identified five *de novo* induced compounds, and the structure of one was successfully achieved (4′-hydroxysulfoxy-2,2′-dimethylthielavin P).

Bacterial mixed cultures that led to the synthesis of previously unknown SMs mostly involve gram-positive bacteria, such as streptomycetes, which form the largest genus in the actinomycetes order and represent an unlimited source of novel compounds, including many therapeutic molecules with anti-tumor, anti-cancer, antibiotic, and antifouling properties (Subramani and Aalbersberg, 2012; Doroghazi et al., 2014). As reported, *Streptomyces lividans* TK23 produces a red pigment after the direct interaction with the mycolic acid-containing bacterium *Tsukamurella pulmonis* TP-B0596 (Onaka et al., 2011). In parallel, *T. pulmonis* TP-B0596 is also able to induce natural product synthesis or, at least, to enhance their production in other *Streptomyces* strains. Accordingly, a novel antibiotic named alchivemycin A was isolated from the culture broth of the co-cultivation between *T. pulmonis* and *Streptomyces endus* (Onaka et al., 2011).

Streptomycetes are not only soil microorganisms, but are also widespread throughout marine ecosystems and have been isolated from various seaweed and marine sediments. Co-cultivation of marine streptomycetes was also successfully used to encrypt silent gene clusters. They have also been found to represent a promising source of antifoulants (Xu et al., 2010). Biofouling, the accumulation of microorganisms, algae and plants on wet surfaces, is one of the most serious problems encompassed in various marine industries. The active antifouling diterpene lobocompactol was rapidly induced and isolated from the marine actinomycete *Streptomyces cinnabarinus* (PK209) after co-cultivation with the lobocompactol-resistant bacterium KNS-16 (*Alteromonas sp.*; Cho and Kim, 2012), leading to the isolation of an extremely valuable compound for both marine ecology and industry.

## Induction of Fungal Silent Secondary Metabolite Gene Clusters by Co-Cultivation with Bacteria

In nature, interactions between bacteria and fungi are commonly present. Physical contact between these microorganisms can be assumed in various environments, such as soil, food or even patients (e.g., cystic fibrosis) where organisms can live in close contact and compete for different resources (Frey-Klett et al., 2011). Already in 2001, the production of pestalone, a potent antibiotic against methicillin-resistant *Staphylococcus aureus* (MRSA) and vancomycin-resistant *Enterococcus faecium*, was obtained in the co-culture of a marine-derived gram-negative bacterium of the genus *Thalassopia* sp. (CNJ-328) and the marine fungus *Pestalotia* (Cueto et al., 2001). Although fungi-bacteria consortia exist in both terrestrial and marine environment, the majority (>90%) of the currently known microbial natural compounds are derived from terrestrial microorganisms (Subramani and Aalbersberg, 2012). *Streptomyces*, *Pseudomonas*, and *Bacillus* are reported to be the most commonly found bacteria in soil and the rhizosphere (Bouizgarne, 2011) and play the most important role as fungal partners. The gram-positive model organism *Bacillus subtilis* is one of the most frequently found microorganisms in the rhizosphere. It can also induce SM production in fungi, as proved by the formation of macrocarpon C, 2-(carboxymethylamino)benzoic acid and (–)-citreoisocoumarinol in *Fusarium tricinctum* (Ola et al., 2013). Compared to the fungal monoculture, the production of lateropyrone, cyclic depsipeptides of the enniatin type, and fusaristatin A were up to 78-fold higher (Ola et al., 2013). Also marine-derived fungal-bacterial communities have been found to be a promising origin of novel SMs (Marmann et al., 2014). Oh et al. (2007) observed that co-cultivation of a marine fungus identified as *Emericella parvathecia* and the actinomycete *Salinispora arenicola* led to a 100-fold production of emericellamides A and B by the fungus. Both metabolites showed a slightly increased activity against MRSA. *Emericella* is the teleomorph (sexual form) of many *Aspergillus* spp. (Geiser, 2009). In fact, the emericellamide biosynthetic gene cluster, which contains a PKS and a NRPS, was also described in the model organism *A. nidulans* (Chiang et al., 2008). Co-cultivation of the marine α-proteobacterium *Thalassopia* sp. (CNJ-328) and the fungus *Libertella* sp. led to the production of libertellenones A-D by the fungus. A direct physical contact appears to be important for libertellone production, as the diterpenoids were neither produced in a *Libertella* monoculture nor by adding supernatant or extract of the bacterial culture (Oh et al., 2005). Libertellenones showed an increased cytotoxic activity against human adenocarcinoma cell line (HCT-116), but no antibiotic properties.

*Aspergillus fumigatus*, the most common airborne fungal pathogen, has been reported to produce at least 226 potentially bioactive SMs (Frisvad et al., 2009) including well studied metabolites like gliotoxins, pseurotins, and fumagillins. Again, most of the biosynthetic gene clusters are silent under laboratory conditions. Zuck et al. (2011) co-cultured *A. fumigatus* with *Streptomyces peucetius* which led to the formation of formyl xanthocillin analogs, named fumiformamide, and *N,N′-*((1Z,3Z)-1,4-bis(4-methoxyphenyl)buta-1,3-diene-2,3-diyl)diformamide. *A. fumigatus* co-cultured with *Streptomyces bullii* produced ergosterol and numerous new metabolites, including seven metabolites of the diketopiperazine alkaloids, brevianamide F, spirotryprostatin A, 6-methoxy spirotryprostatin B, fumitremorgin C and its 12,13-dihydroxy derivative, fumitremorgin B as well as verruculogen, 11-*O*-methylpseurotin A and a new isomer 11-*O*-methylpseurotin A_2_ (Rateb et al., 2013). *A. fumigatus* is also part of a microbial interaction in another unusual habitat—coal mine drainages where such interactions may be helpful for survival. Co-cultures of two coalmine drainage-derived organisms, a *Sphingomonas* strain and an *A. fumigatus* strain led to the detection of glionitrin A, which is a new diketopiperazine (Park et al., 2009). Glionitrin A shows significant antibiotic activity against both MRSA as well as increased cytotoxic activity against four human cancer cell lines. Further potential microbial interactions were revealed in the genus *Fusarium*, which are also filamentous fungi widely distributed in the soil. Analysis of the interaction between *Fusarium pallidoroseum* and *Saccharopolyspora erythraea* resulted in three new decalin-type tetramic acid analogs related to equisetin (Whitt et al., 2014).

## Functional Analysis of Microbial Communication

The various examples presented above illustrate that mixed microbial fermentations are an emerging field in microbiology. They can be seen as a strategy to mimic the physiological conditions in the different microbial consortia. The better understanding of the native bacterial-fungal interactions will not only expand our possibilities to identify interesting new SMs (e.g., lead structures), but also affect our knowledge on how these consortia are structured by the signals derived from the involved species. In a recent study it was shown how SMs contribute to the structure of microbial communities (Donia et al., 2014). The biosynthetic capacity of the human microbiome was explored by systematic analysis of its biosynthetic gene clusters and identified the thiopeptide lactocillin, which is produced by the vaginal commensal *Lactobacillus gasseri*. Interestingly, lactocillin is active against several pathogens like *S. aureus* and *Corynebacterium aurimucosum*, but inactive against commensals thus influencing the microbial composition of this specific habitat. Metatranscriptomic data analysis revealed that the corresponding thiopeptide biosynthetic gene cluster is indeed expressed *in vivo* in human samples (Donia et al., 2014). Something similar was shown in other kingdoms. In another example the effect of SMs produced by endophytic fungi on the cohabitating host plant was shown to provide benefits to the host. In mixed microbial cultures the endophytic fungus, *Alternaria tenuissima*, significantly increased the production of several polyketides, including the antifungal stemphyperylenol, which is active against another endophytic fungus, *Nigrospora sphaerica*, a well-known leaf pathogen.

True symbioses between microorganisms have even shown a fruitful source for new SMs. A very special kind of interaction between a fungus and a bacterium is that of the zygomycete *Rhizopus microsporus* harboring endosymbiotic bacteria of the species *Burkholderia rhizoxinica*, a novel species discovered by Partida-Martinez et al. (2007a). Together with its symbiont the fungus is an important plant pathogen causing rice seedling blight. For more than two decades, it was thought that the fungus produces the causal agent for the plant disease. As shown, the endosymbiont is the actual producer of the phytotoxin, rhizoxin, that binds to the β-tubulin of the rice plant cells and causes mitotic arrest (Partida-Martinez and Hertweck, 2005, 2007; Partida-Martinez et al., 2007b). This, in turn induces the typical symptoms of swelling of the seedling tips and finally resulting in the death of the plants’ offspring (Scherlach et al., 2006). Additionally, it has also been shown that the endobacterium is obligatory for sporulation of its host fungus (Partida-Martinez et al., 2007b). Elucidation of the underlying molecular mechanisms of this interaction (Leone et al., 2010) led to the discovery of “self” resistance mechanisms of the fungus against the mycotoxin (Schmitt et al., 2008) and of factors essential for symbiosis (Leone et al., 2010; Lackner et al., 2011). Recent data revealed that a type 2 secretion system (T2SS) is also required for the formation of the endosymbiosis between the fungus and the endobacterium (Moebius et al., 2014). By use of comparative proteome analysis, it was shown that chitinolytic enzymes and chitin-binding proteins were released by the secretion system of the bacterium. Further experiments (e.g., targeted gene-knock-outs, sporulation assays) clearly showed that a chitinase is essential for the bacteria to enter the hyphae (Moebius et al., 2014). More recently, the biosynthesis of antifungal and antibacterial polyketides by *Burkholderia gladioli* in co-culture with *R. microsporus* has been investigated (Ross et al., 2014). Conditions emulating tempe bongkrek production, a type of fermented soybeans made with the addition of coconut, resulted in the formation of novel members of the enacyloxin family of antibiotics and to enhanced production of the toxin, bongkrekic acid, by the tempe contaminant *B. gladioli*.

Overall, the underlying mechanisms of SM biosynthetic gene cluster regulation are emerging, but are still poorly understood. Only few studies reported the gene regulation mechanisms involved in SM formation during microorganism interaction. One of them is the antibiotic concanamycin A production by *Streptomyces halstedii*. Concanamycin A alters the proteomic profile of *A. nidulans* and probably plays an active role in defense-related pathways (Melin et al., 2002). Another example, which will be extensively described below, is the specific interaction between *A. nidulans* and *S. rapamycinicus*. During this mutual interplay, the activation of silent gene clusters, and subsequent production of novel compounds, is transduced by affecting the chromatin-based regulation in the eukaryotic partner (Schroeckh et al., 2009; Nützmann et al., 2011).

## The Interaction of *Aspergillus* with *Streptomyces rapamycinicus*

It was discovered that the intimate physical contact of *A. nidulans* with a distinct soil-dwelling bacterium, *S. rapamycinicus*, identified from a collection of 58 species of actinomycetes, led to the selective activation of silent PKS and NRPS gene clusters in the fungus (Schroeckh et al., 2009). One induced cryptic PKS gene encodes the long sought-after orsellinic acid synthase, thus the corresponding cluster was named the *ors* gene cluster. In addition to this archetypal polyketide orsellinic acid, three derivatives (lecanoric acid and two cathepsin K inhibitors F-9775A and F-9775B) were produced by *A. nidulans*. Lecanoric acid is a typical lichen metabolite that is usually found in a fungal/bacterial mutualism (Stocker-Worgotter, 2008), and thus likely plays a role in microbial communication. Indeed, the inducing bacterium was not affected by lecanoric acid. As mentioned above, a physical contact between both partners is needed for the activation of this silent gene cluster (Scherlach and Hertweck, 2009; Schroeckh et al., 2009). It is conceivable that a symbiotic relation between the fungus and the bacterium exists to defend against other microorganisms. One explanation on how the bacterium can trigger SM formation in *Aspergillus* would have been that rapamycin produced by the streptomycete could activate the cluster, either *via* the inhibition of the TOR pathway (Fitzgibbon et al., 2005) or due to its more general antifungal activity. Alternatively, another bacterial metabolite, the fungistatic antibiotic trichostatin A (TSA), which is produced by *Streptomyces hygroscopicus* could be responsible *via* its respective histone deacetylase (HDAC) inhibiting activity (Tsuji et al., 1976; Kouraklis and Theocharis, 2002). However, neither the addition of rapamycin nor TSA led to the activation of the *ors* gene cluster, therefore making both compounds unlikely to play a role in the interaction. When *S. rapamycinicus* was co-cultivated with *A. fumigatus*, this fungus also displayed an altered SM profile showing a group of similar new SMs (König et al., 2013). In a microarray approach, a SM gene cluster was identified that was up-regulated only in the co-culture. Deletion of the PKS of the identified cluster, correlated with the lack of the corresponding natural products. Two metabolites of this group were isolated and named fumicycline A and B and the corresponding PKS was designated as FccA. It was shown that again a direct physical contact was necessary to induce the *fcc* gene cluster. An ortholog of the *fcc* gene cluster was identified in *Neosartorya fischeri* (Chooi et al., 2013). Overexpression of the transcription factor gene of the cluster led to the production of neosartoricins. These metabolites demonstrate high similarity to fumicyclines and showed T-cell antiproliferative activity, suggesting a physiological role as an immunosuppressive agent (Chooi et al., 2013).

**FIGURE 2:**
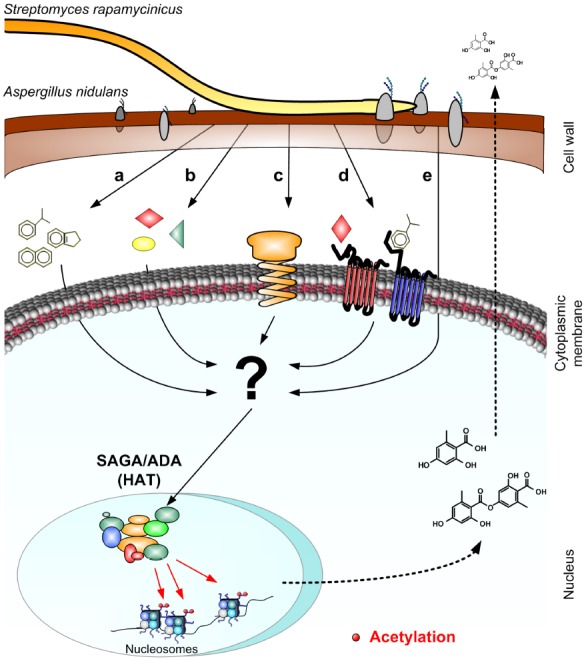
**Model of the interaction between *A. nidulans and S. rapamycinicus*.** The figure presents hypotheses about different stimuli that could be responsible for the activation of SM gene clusters during the interaction between *A. nidulans* and *S. rapamycinicus*. The signal that finally results in the Saga/Ada complex activation could derive from at least five possible events: possibly membrane-bound compounds (a), or peptides (b), could reach the cytosol of the fungus and indirectly activate the Saga/Ada complex. Alternatively, a specific fungal receptors could recognize either the attachment of the bacterium (c), or compounds secreted during the interaction (d). A further hypothesis could be that fungal surface proteins could recognize the streptomycete directly triggering a signaling cascade (e). The internal signal that should lead to the activation of the Saga/Ada complex is unknown.

Studies of various chemical inhibitors led to the striking finding that the interaction between *S. rapamycinicus* and *A. nidulans* relies largely on the activity of chromatin remodelers. Supplementation of the co-culture with a TSA-like HDAC inhibitor, suberoylanilide hydroxamic acid (SAHA), and with the histone acetylase (HAT) inhibitor anacardic acid led to the activation and inhibition of the transcription of the *ors* gene cluster, respectively. These findings indicated that chromatin remodeling can play an essential role in the regulation of SM clusters and that the targeted activation or inactivation of the respective chromatin modifiers can alter the SM production of the fungus. Nützmann et al. (2011) demonstrated that acetylation plays an essential role for mediating the interaction. Therefore, a comprehensive deletion library of all putative HATs in *A. nidulans* was generated and systematically screened for the ability of mutants to activate the *ors* gene cluster during co-incubation with *S. rapamycinicus* (Nützmann et al., 2011). Thereby, the HAT GcnE was identified as being essential for the cluster induction. This HAT is the catalytic subunit of the Saga/Ada complex (see Figure 2), a conserved multi-subunit complex also found in other eukaryotic organisms (Baker and Grant, 2007; Govind et al., 2007). Furthermore, it was shown that the acetylation of histone H3 lysines 9 and 14 is needed for the onset of the *ors* gene cluster transcription and product formation (Nützmann et al., 2013). However, SAGA not only seemed to play a role during the interaction with *S. rapamycinicus*, but also for the regulation of other well-known natural products such as penicillin, sterigmatocystin and terrequinone A in *A. nidulans* (Nützmann et al., 2011, 2013). Due to the intimate contact of *S. rapamycinicus* with *A. nidulans* the question arose whether there is a common mechanism by which the bacterium might interact with other members of the *Aspergillus* family, e.g., with *A. fumigatus*. It is possible to postulate different ways that can lead to the activation of such clusters in the fungus. As shown in Figure 2, an unknown, possibly membrane-bound compound or a protein can modulate the Saga/Ada complex directly (Figures 2a,b). Alternatively, the signal could be induced either by the physical contact between the two organisms (Figure 2c), or by a protein or compound secreted by the bacterium, and specifically sensed by receptors of *A. nidulans* (Figure 2d). These components need to be specific for *S. rapamycinicus* and must not be found in other actinomycetes. Convincible is also that the recognition of a fungal surface protein by the streptomycete, could directly lead to the activation of a signaling cascade triggering the SAGA complex (Figure 2e). In the interaction of *A. nidulans* with *S. rapamycinicus* the *ors* gene cluster regulation relies largely on the activity of GcnE and its acetylation activity of lysine 9 and 14 at histone H3. This in turn is induced upon physical contact with the bacterium leaving room for speculation on the key influence of the streptomycete on the fungus. Regarding the signaling pathway behind this interaction, it is known that LaeA as a global SM regulator has no influence on GcnE and therefore on histone H3 acetylation (Nützmann et al., 2011). This means that there must be an alternative pathway and a transcriptional regulator responsible for the recruitment of the HAT and the multi-subunit complex, SAGA, to the respective loci.

For some clusters, such as penicillin, it has already been shown that some general regulators are required like the major pH regulator PacC, which activates penicillin biosynthesis at alkaline pH in *A. nidulans*, or the CCAAT binding complex (Tilburn et al., 1995; Litzka et al., 1998; Then Bergh and Brakhage, 1998). For the *ors* gene cluster, however, the key regulators still remain to be discovered.

## Modulation of Gene Expression by Interaction Partner Induces Chromatin Remodeling

The impact of chromatin remodeling on the communication between organisms or the control of host gene expression has gained attention in the last years. There are several examples of bacterial pathogens interfering with the host regulatory system of gene expression. Fewer are known about the regulatory mechanisms of interactions involving fungi, especially when it comes to those leading to the expression of SM gene clusters. However, some light has been shed on the impact of chromatin remodeling on natural product regulation in fungi. By now, a variety of chromatin modifiers have been discovered, which regulate SM biosynthesis in filamentous fungi (Gacek and Strauss, 2012). Most knowledge so far has been gained on acetyltransferases (HATs), which are grouped in diverse families, comprising amongst others the MYST-family, p300/CBP-family, and the Gcn5-related acetyltransferases (GNATs) (Carrozza et al., 2003). The latter includes Gcn5, the catalytic subunit of the SAGA complex, also referred to as GcnE in *Aspergillus* species. As mentioned before, a distinct deletion mutant of the knock-out library of HATs in *A. nidulans* led to an altered SM biosynthesis pattern. Hence, it was speculated that the systematic screening of the deletion library allows for the identification of novel metabolites. Indeed, a drastically altered metabolic profile was detected in the Δ*nnaB* (nidulans *N*-acetyltransferase B) deletion mutant. Aside from a number of orsellinic acid derivatives, there was also a new class of compounds identified as pheofungins, which are heterocyclic molecules with cytotoxic activity (Scherlach et al., 2011). HAT modification led the production of SMs also in other fungi. The aflatoxin biosynthetic gene cluster in *Aspergillus parasiticus* was also shown to be co-regulated by a MYST-type family member of the HATs. Furthermore, the transcription of the aflatoxin cluster genes coincides with the acetylation of histone H4, showing that a HAT is involved in the regulation of this cluster (Roze et al., 2007, 2011). Soukup et al. (2012) obtained similar results by overexpressing *esaA*, a gene encoded for a MYST-type HAT. The overexpression of this gene affected the production of penicillin, sterigmatocystin, terrequinone A, and the *ors* gene clusters in *A. nidulans* (Soukup et al., 2012).

The example of *S. rapamycinicus*, which mediates cluster regulation in *A. nidulans via* an increased histone acetylation upon contact, shows how bacteria can interfere with the eukaryotic histone modification system (epigenetic regulation). Other bacteria were also reported to have a similar impact on eukaryotic cells. *Listeria monocytogenes*, the producer of the toxin listeriolysin O, is a bacterium causing foodborne infections leading to sepsis, miscarriages during pregnancy, and meningitis, and is largely found in immunocompromised patients. Hamon et al. (2007) were able to show that listeriolysin O caused a dramatic modulation of the host gene expression. This was caused by deacetylation of histone H4 but also drastic dephosphorylation of serine 10 on H3 thus leading to a downregulation of substantial immunity factors in the host cells. Similar observations have been made for *Clostridium perfringens* and *Streptococcus pneumoniae*, the producers of perfringolysin and pneumolysin, respectively (Maekita et al., 2006; Hamon et al., 2007). Both toxins also led to dephosphorylation of the host’s chromatin. Thus, different toxins secreted by bacteria appear to manipulate and control chromatin remodeling and thereby transcription of targeted genes of eukaryotic hosts.

The so-called erasers of acetylation are the HDACs that fulfill the opposing reaction of the HATs by removing the acetyl group from lysine residues of the histone proteins. HdaA, a class II HDAC, was one of the first discovered to play a role in SM cluster regulation. The deletion of *hdaA* in *A. nidulans* not only led to reduced growth of the fungus during oxidative stress, but also resulted in a higher production of SMs such as penicillin, sterigmatocystin, and terrequinone A (Tribus et al., 2005; Shwab et al., 2007). Consistently, HdaA had a significant impact on SM produced in *A. fumigatus*, such as fumitremorgin B, pseurotin, and gliotoxin. Interestingly, however, was the finding that gliotoxin production was down-regulated upon deletion of *hdaA* in *A. fumigatus* (Shwab et al., 2007; Lee et al., 2009). Garcia-Garcia et al. (2009) were able to connect the activity of the human HDAC1 with the infection process of *Anaplasma phagocytophilum* in THP-1 cells (granulocyte model). Hereafter, the infection process led to an increased activation of HDAC1 leading to a reduced histone H3 acetylation and to the silencing of host defense genes. In accordance, the inhibition of HDAC1 by siRNA led to a significant drop of the bacterial load. This shows that the epigenetic control of the host cell by the bacterium promotes the disease by increased survival of the pathogen (Garcia-Garcia et al., 2009).

Besides the widely studied acetylation of histones, there is a multitude of other chromatin modifications which have been found to regulate expression of SM gene clusters (Strahl and Allis, 2000). Methylation of lysine is regarded as one of the most complex modifications found so far with diverse impacts on gene transcription depending on its conformation (Rolando et al., 2013). Reyes-Dominguez et al. (2010) showed that upon growth arrest the methylation of lysine 9 was subsequently reduced, but affected only genes located inside the sterigmatocystin cluster, leading to its activation. Furthermore, H3 K9 methylation marks were associated with heterochromatin protein A (HepA), a protein responsible for heterochromatin formation. Consistently, the deletion of HepA led to the activation of the *stc* gene cluster (Reyes-Dominguez et al., 2010; Brakhage, 2013). However, the combination of both the *hepA* and the *laeA* deletions reduced the sterigmatocystin production to wild-type levels (Shaaban et al., 2010). The global SM regulator LaeA was indirectly found to be involved in histone methylation by influencing the methylation of H3 K9 and the occupancy of the respective locus by HepA. The deletion of this gene was also found to constrain the expression of the prominent natural product gene clusters penicillin, sterigmatocystin and the cholesterol lowering agent lovastatin (Bok and Keller, 2004; Reyes-Dominguez et al., 2010). In another study, the heterochromatin protein, HP1 of the fungus *Leptosphaeria maculans*, could be implicated in the pathogenicity process responsible for plant infection. The fungus harbors effector genes with low expression during axenic cultivation, while being highly transcribed upon co-cultivation with plants. In an infection model with *Brassica napus* the effect of histone H3K9me3 on the respective effector genes was investigated. HP1 as well as the DNA-methylase DIM-5 were silenced by RNAi and analyzed concerning the transcription level of the effector genes in axenic cultures. Interestingly, the effector genes were actively transcribed in the mutant strains outside of the co-cultivation leading to the conclusion that HP1 as well as DIM-5 must be somehow involved in the repression of the effector genes during the non-infective life cycle (Soyer et al., 2014). Additionally, in a symbiotic interaction of the endophyte *Epichloë festucae* and *Lolium perenne*, the fungus produced ergot alkaloids and lolitrems when cohabitating with a plant. Production of these bioprotective substances was repressed during axenic cultures. Comparing levels of H3K9me3 and H3K27me3 between co-cultivation and non-symbiotic cultivation of the fungus, the methylation marks were reduced upon growth in the plant. Furthermore, the deletion of the responsible methyltransferases ClrD and EzhB resulted in an activation of the alkaloid and lolitrem gene clusters also in the axenic cultures of the fungus (Chujo and Scott, 2014).

Methylation of lysines 4 at histone H3 by CclA was found to be important for SM biosynthesis as well as for conidiation in *A. nidulans* and *A. fumigatus* (Palmer et al., 2008, 2013; Bok et al., 2009). The deletion of the respective genetic locus in *A. nidulans* not only led to the production of F-9775A and F-9775B, which are also produced upon contact with the bacterium *S. rapamycinicus*, but led also to the activation of a novel monodictyphenon gene cluster. Another very interesting study was published by Rolando et al. (2013). They showed that pathogens can also introduce, so far, unknown modifications on host nucleosomes and thereby influence gene expression. In their study they elegantly revealed that *Legionella pneumophila* is able to tri-methylate lysine 14 on histone H3 of its host by a factor called RomA. This protein is a SET-domain containing methyltransferase which is secreted by *Legionella* during the infection process. Genome-wide ChIP analysis showed that approximately 4870 promoters were target of this modification by RomA (Rolando et al., 2013). The cause for this drastic modulation of the host genome by *Legionella* is not fully understood yet. One possible explanation might be that the switch to a methylated histone leads to down-regulation of the target genes due to a mutual exclusion of the acetylated lysine 14, which was found to coincide with actively transcribed genes (Cheung et al., 2000).

In summary, the impact of post-translational regulation on SM cluster expression and the interaction of organisms have revealed their great potential for future studies in natural product research. Prokaryotes are able to modify their host’s gene expression in multiple ways. Bacterial toxins were shown to be useful tools during the infection process by reducing levels of acetylation and phosphorylation of histones and thereby influencing the expression of their target genes. Often, chromatin modifying complexes are mediators of those interactions, specifically targeting host defense genes and modulating their expression. Interestingly, this is not only achieved by host derived remodelers but also by proteins introduced by the interaction partner itself, which in turn can lead to unknown modification on the host genome. Taken together, recent studies have shown the great potential of bacteria and fungi to modulate gene expression of organisms during co-cultivation experiments. Seeing this, it is convincible that the investigation at the molecular basis of multispecies interaction has great potential. The more we understand about communication between species, the better we can trigger the discovering of unknown natural products in microorganisms.

## Perspectives for the Analysis of Natural Products

**FIGURE 3:**
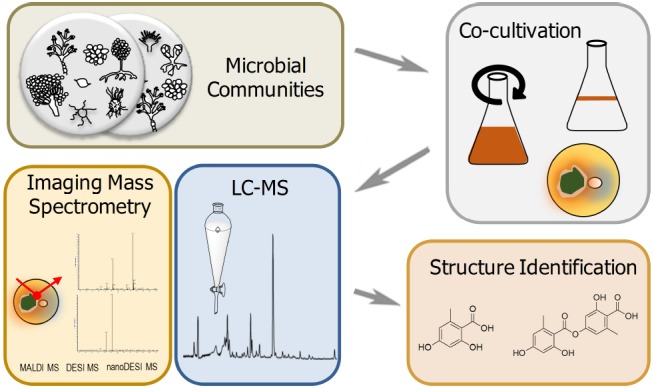
**Proceeding of natural product analysis from microbial co-cultivations.** Microbial communities are co-cultured in flasks (static or planktonic) or on plates (solid-state culture). Primary analysis of natural products via extraction and LC–MS methods or directly evaluated by imaging mass spectrometry based methods. Subsequent structure identification of unknown masses can be conducted using NMR techniques.

In the search for SMs in co-cultivations, one must also determine which analytical method to use for the detection of these compounds. This topic has already been extensively reviewed by other groups (Scherlach and Hertweck, 2009; Tarkka et al., 2009; Bertrand et al., 2014b; Marmann et al., 2014; Schroeckh et al., 2014), but the most interesting new studies will be covered here. Thus far, the methods for natural product analysis have ranged from simply the extraction of co-cultures in liquid/solid media to the use of quite novel techniques such as imaging/real-time mass spectrometry that can be carried out in solid-state cultures. The former technique has shown to be of value resulting in the discovery of many new SMs or in the study of the regulation of different products, which cannot be found in monocultures. This technique commonly entails the extraction of the natural products from the culture broth, which are then subjected to a form of liquid chromatography-mass spectrometry (LC-MS). In a further step, potential new SMs can be purified and isolated for structural elucidation by nuclear magnetic resonance (NMR) spectroscopy (Figure 3). This workflow was applied to the discovery of new SMs from the co-cultivation of *S. rapamycinicus* with *A. nidulans* and *A. fumigatus*, respectively. In both cases new fungal products were detected by LC-MS when the streptomycete was added (see above). Other examples for LC-MS detection of co-culture-derived products are the new antibiotic alchivemycin A in *S. endus* by the mycolic acid-containing bacteria *T. pulmonis* (Onaka et al., 2011; see above), as well as the co-culture of *Streptomyces coelicolor* with myxobacterium *Corallococcus coralloides*, where the streptomycete increased the production of the biologically active compound undecylprodigiosin 60-fold (Schäberle et al., 2014). Because of its potential for the discovery of new natural products, there is also a need for high-throughput methods to encompass large-scale co-cultivations. This question was addressed for the study of more than 600 different fungal strains. With the help of automated data analysis, new molecular masses were observed which were not found in natural product databases (Bertrand et al., 2013a). In a further example of high-throughput screening, fungal co-cultures were cultivated with very small culture volumes. A big advantage of small culture volumes is that sample preparation can be completed in less time and the number of different cultures can be increased (Bertrand et al., 2014a). These are just few examples to show that LC-MS analysis of co-cultivations can be a very practical tool, and because of the constant problem to obtain enough product for structure elucidation, scale-up of these cultures can usually be accomplished.

Besides the well-established exploration of co-cultures for new bioactive compounds by LC–MS, there has also been advancements in the field of imaging and real-time mass spectrometry (Fang and Dorrestein, 2014), where metabolites can be detected by their spatial distribution. Imaging mass spectrometry (IMS) has, for the most part, been associated with matrix-assisted laser desorption/ionization (MALDI), which is then coupled to a mass spectrometer, for which images can be produced depicting the spatial organization of natural products (Cornett et al., 2007; Esquenazi et al., 2009; Watrous and Dorrestein, 2011; Bouslimani et al., 2014; Shih et al., 2014). This technique has shown to be useful in observing the role of natural products in the interaction between different microorganisms, such as *A. fumigatus* with *Pseudomonas aeruginosa* (Moree et al., 2012), *B. subtilis* with *S. coelicolor* (Yang et al., 2009), *B. subtilis* with *S. aureus* (Gonzalez et al., 2011), and the cannibalism of *B. subtilis* (Liu et al., 2010), just to name a few. Additionally, two promising examples, which aided in the discovery of novel natural products by IMS will be discussed. The first demonstrated that the infection of the button mushroom, *Agaricus bisporus*, with the soft rot-causing bacterium *Janthinobacterium agaricidamnosum* and the brown blotch disease-causing bacterium *Pseudomonas tolaasii*. *J. agaricidamnosum* infected mushrooms revealed the presence of a novel virulence factor, jagaricin when analyzed by MALDI-MS at the sites of infection. This substance was shown to play an important role in soft rot of mushrooms and also appeared to be a potent antifungal (Graupner et al., 2012). The second example, also using IMS, investigated the infection of *A. bisporus* with *P. tolaasii* and could show that the tolasin metabolites, which were observed at the site of infection, are responsible for this disease (Scherlach et al., 2013).

Similarly, a more recently developed technique, real-time mass spectrometry, encompassing the techniques of desorption electrospray ionization (DESI) or nanospray desorption electrospray ionization (nanoDESI), is also a sufficient option in detecting natural products in co-cultivations. An advantage of this method compared to MALDI, is that it does not depend on the formation of the matrix and also has little to no sample preparation. Measurements can be taken directly from the plate and can also be used for IMS. Moreover, the method is usually termed ambient mass spectrometry because ionization takes place at atmospheric conditions and room temperature. For further information the following reviews are recommended (Bouslimani et al., 2014; Fang and Dorrestein, 2014; Hsu and Dorrestein, 2015). Furthermore, Watrous et al. (2013) have also shown a methods paper using nanoDESI IMS with little sample preparation of different microbial monocultures and co-cultures directly from a Petri dish. nanoDESI IMS has even led to the discovery of new desferrioxamine derivatives in co-cultures of *S. coelicolor* with other actinomycetes (Traxler et al., 2013). The use of IMS for the detection of natural products is an ever-evolving field and new techniques are constantly being discovered and older techniques optimized. One recent ionization method, direct analysis in real time mass spectrometry (DART-MS), could also be used in studying the role of SMs in co-culture (Gross, 2014).

## Conclusion

Secondary metabolites are of major interest due to their applicability as therapeutic agents. To satisfy the constant need for new SMs, and to come up against the continuous emerging of bacterial resistant strains, it would be advantageous to understand the SMs’ physiological relevance and their ecological significance. In this context, mixed microbial cultivations have become a powerful method to induce previously unexpressed biosynthetic pathways, leading to the production and identification of new SMs (Schroeckh et al., 2009; Bertrand et al., 2014b; Marmann et al., 2014). A greater understanding of the underlying molecular mechanisms driving microbial co-cultivations would be important for deriving general mechanisms. This knowledge could be used specifically to induce silent SM biosynthesis gene cluster in laboratory conditions. However, a deeper understanding of the SM biosynthetic gene cluster regulation alone will not be sufficient. Due to the often very tiny amounts of SMs produced by the microorganisms, the development of analytic tools is getting more and more important. At the same time, multidisciplinary collaborations are necessary ensuring a careful analysis and validation process of the collected data from any MS method in terms of dereplication (Hufsky et al., 2014). Therefore, data collection and processing could be applied in global libraries, as seen for genome and transcriptome data, and used to help the scientific community in the constant race between the discovery of new antibiotics and the continuous emergence of resistance mechanisms.

### Conflict of Interest Statement

The Guest Associate Editor Nancy Keller declares that, despite having collaborated with author Axel A. Brakhage, the review process was handled objectively and no conflict of interest exists. The authors declare that the research was conducted in the absence of any commercial or financial relationships that could be construed as a potential conflict of interest.
